# The Use of Steam in Diseases of the Uterus

**Published:** 1897-01

**Authors:** H. Leonards

**Affiliations:** New Braunfels, Texas


For the Texas Medical Journal.

### THE USE OF STEAM IN DISEASES OF THE UTERUS.

BY H. LEONARDS, M. D., NEW BRAUNFELS, TEXAS.

[Read before the Austin District Medical Society, December 24, 1896.]

PROF. SNEGIROFF, of Moscow, last year published an article in the *St. Petersburg Medical Journal* on the treatment of uterine hemorrhage by steam. The remarkable success of this agent in the hands of this author induced me to give it a thorough trial.

I shall not make any distinction in this consideration between menorrhagia and metrorrhagia, nor between hemorrhages based on endometritic fungosities, and such due to neoplasms, as carcinoma or fibroids, etc., as in all these conditions I deem the steam application a potent factor for the arrest of hemorrhage.

I have, up to date, treated nine cases of uterine hemorrhage with steam, and the result was far above my expectation in every case. In two of these cases, curettage was resorted to shortly before, resulting in the arrest of hemorrhage for only ten days. After one application of steam, the hemorrhage ceased permanently.

Concerning the technique of this treatment, I need but briefly dwell upon it. After some experimenting, I succeeded in constructing a compact and simple apparatus, which meets all requirements. A small steam kettle, heated by an alcohol lamp, is connected with a male silver catheter, by means of a rubber tube two feet in length. The catheter differs from an ordinary one in having at its end, instead of two large eyes, twenty or thirty small perforations, evenly distributed over its last two inches. Before I bring my patient into position, I light the alcohol lamp, and place the apparatus to my right, within convenient reach. After introducing a Jacob's weight speculum, I grasp the cervix with a vulsella forceps. If the cervix is very narrow, it must be dilated. Meanwhile, the water in the kettle is boiling, and the steam is escaping through the perforated catheter. I introduce the instrument into the uterus, and retain it there for about two minutes. After removal of the catheter, I swab the vagina with cotton, and the whole procedure is completed. Antiseptic preparatory measures are unnecessary, as the overheated steam is the best antiseptic itself. The after-treatment consists of a daily vaginal douche. The operation is painless, where care is taken that the catheter does not come in contact with the vaginal wall.

As stated above, I have treated nine cases of uterine hemorrhage in this manner, the result of which leads me to believe that the steam treatment is superior to any other. It is surer, and more prompt in its action; more simple and less dangerous than all the other methods, not excepting tamponade or curettage of the uterus.

Some months ago, Dr. Punecki, of Danzig, published an article on the treatment of chronic endometritis with steam. After a trial in thirty cases, he came to the conclusion that this treatment gives better results than any other now in vogue.

The treatment of endometritis chronica consisted, up to the present time, in some application to the diseased endometrium, the application of caustics in substance (argent. nitr., cupr. sulph., ferri sesqui.), the injection of caustic fluids, tinct. iodine, etc.; the brushing with different chemicals, such as ichthyol, thimol, chloride of zinc, etc.; the galvano-chemical current, and the curettage. All these methods have but one end in view, namely, the necrotizing of the endometrium, so that it may be replaced by a new and normal one, growing from the deeper cells out of the glandular ducts. Taking this into consideration, it is evident that the application of steam should recommend itself in the treatment of chronic endometritis as a logical one.

During the last six months, I had the good fortune of trying the steam treatment in five consecutive cases of chronic endometritis. The treatment consists in applying the steam for about two minutes every two weeks, three or four consecutive times. The result in every instance was most satisfactory. The secretion changed in quality and quantity, and after three applications, seemed to be quite normal. One of these cases proved to be of special interest, as not only the endometritis was favorably influenced, but the co-existing chronic metritis also. The uterine cavity, which, in the beginning, showed a depth of 5½ inches, gradually diminished with each application, so that after the third injection it is only 3¼ inches. A similar case, which is at present under my care, promises equally well, judged by the first application. If this experience should be corroborated by future trials, great progress will, no doubt, be insured in dealing with such cases. The present treatment of chronic metritis with voluminous hot water injections, or with glycerine-ichthyol tampons, etc., is certainly so slow and disagreeable that any new line of treatment ought to be welcome to the physician.

From the limited experience I have had in the treatment of uterine hemorrhage and chronic endometritis with steam, I feel justified in recommending this therapeutic measure to the profession for future trial. Physicians who have to deal with such cases in general practice, know how few satisfactory results are obtained from the customary line of treatment, not to speak of the occasional accidents, severe colics, etc. Curettage, which is the favored treatment of most gynecologists, must be looked upon as an operation of some gravity, and requires minute preparatory arrangements, like other serious operations. It can not be performed in an ambulatory way, nor does it permit the patient to follow her daily avocations. There are many cases on record, and surely many more not published, where curettage was the starting point of grave peri or parametritic inflammation, or even of fatal peritonitis.

A further point which should be appreciated very highly, is the easiness and simplicity of the procedure. No preparatory measures, no anæsthesia, no assistance, and even no special after-treatment are required. If I am not mistaken, I predict that steam will have an acknowledged place in the therapeutics of said affections. It may supplant the caustic treatment, and even do away with the curette.

On the use of this agent in the various pathological affections of the uterus other than those mentioned, I cannot speak from personal experience. Possibly there may be even a larger field for it. In fibroids and myomata of the uterus, it will find an indication for the arrest of the accompanying hemorrhage. In carcinoma of the uterus, it may be very effective as a disinfectant and deodorant, especially in the last stage, when it becomes very troublesome, on account of the offensive discharge, though I admit that here its real value has yet to be proven by experience.

Finally, I will call the attention of the gynecologist to the value that the steam application may have as an hæmostatic during gynecological operations. I have witnessed several vaginal hysterectomies by competent surgeons, where an insignificant but obstinate hemorrhage caused a delay of half an hour in finishing the operation. The steam would have arrested the hemorrhage immediately, and the patient would have been spared half an honr's prostration from the anæsthesia, which is a significant item in the prognosis of complicated operations. The same may be said of hemorrhages caused by the tearing of extensive adhesions of the intestines. This applies, of course, only to capillary hemorrhage, and to such of small arteries.

The tissues, even the serosa of the gut, bear the application of steam very well. Wounds treated with steam will heal by first intention, which I have proven occasionally in removing a small lipoma. The gut seems to be very resistant to the action of steam. I directed a jet of steam for one minute on one place on the intestine of a little dog without noticing anything unusual.

In giving this communication to the profession, I admit that many points will have to stand the test of experience before their value can find general acknowledgment. My only wish is to draw the attention of my colleagues to this subject, so that they will give it an unbiased trial.

[NOTE.—Dr. Leonard exhibited to the society his devices for administering this treatment, consisting of a self-retaining speculum, and a set of perforated silver catheters, and a steam generotor.—ED.]

---

For the Texas Medical Journal.

